# Neonatal EEG graded for severity of background abnormalities in hypoxic-ischaemic encephalopathy

**DOI:** 10.1038/s41597-023-02002-8

**Published:** 2023-03-10

**Authors:** John M. O’Toole, Sean R. Mathieson, Sumit A. Raurale, Fabio Magarelli, William P. Marnane, Gordon Lightbody, Geraldine B. Boylan

**Affiliations:** 1 INFANT Research Centre, University College Cork, Cork, Ireland; 2 Department of Paediatrics and Child Health, University College Cork, Cork, Ireland; 3 Department of Electronic and Electrical Engineering, University College Cork, Cork, Ireland

**Keywords:** Paediatric research, Biomedical engineering, Hypoxic-ischaemic encephalopathy, Diagnostic markers

## Abstract

This report describes a set of neonatal electroencephalogram (EEG) recordings graded according to the severity of abnormalities in the background pattern. The dataset consists of 169 hours of multichannel EEG from 53 neonates recorded in a neonatal intensive care unit. All neonates received a diagnosis of hypoxic-ischaemic encephalopathy (HIE), the most common cause of brain injury in full term infants. For each neonate, multiple 1-hour epochs of good quality EEG were selected and then graded for background abnormalities. The grading system assesses EEG attributes such as amplitude, continuity, sleep–wake cycling, symmetry and synchrony, and abnormal waveforms. Background severity was then categorised into 4 grades: normal or mildly abnormal EEG, moderately abnormal EEG, majorly abnormal EEG, and inactive EEG. The data can be used as a reference set of multi-channel EEG for neonates with HIE, for EEG training purposes, or for developing and evaluating automated grading algorithms.

## Background & Summary

Impaired oxygen delivery or blood flow to the brain around the time of birth can cause brain injury. Infants can develop an encephalopathy called hypoxic-ischaemic encephalopathy (HIE), which is the leading cause of death and disability in full term neonates. Incidence rates of HIE are around 2 per 1,000 deliveries in high-income countries with higher rates in low- to middle-income countries^1^. HIE can cause neonatal death or significant neurological and neurodevelopmental impairment such as cerebral palsy, epilepsy, or learning disabilities^2^. HIE is an evolving brain injury. The primary injury is followed by a latent phase which lasts for approximately 6 hours. This is followed by the secondary injury phase, a delayed phase of programmed cell death. Therapeutic hypothermia is the only intervention available for infants with moderate to severe HIE and it must be instigated before the onset of the secondary phase of injury if it is to be effective.

The electroencephalogram (EEG) allows for continuous cot-side monitoring of cerebral function. A hypoxic-ischaemic insult can alter the normal background pattern of the EEG, providing a unique insight into cerebral dysfunction^3^. This deviation from normal EEG background is associated with adverse neurodevelopmental outcome^4–6^. As EEG is a valuable measure of severity of ongoing encephalopathy, it can be particularly beneficial when commenced within the primary phase of injury to help determine which infants may benefit from therapeutic hypothermia^7^.

Review of the EEG requires specialist expertise not always available in neonatal intensive care units. Computer-based methods have the potential to automate the process of grading background EEG activity for severity of injury. These automated methods could produce a continuous objective measure of EEG activity that could be easily scaled to monitor a high-volume of neonates, far beyond what would be humanly possible. Many methods have been developed to generate background grading systems^8–17^. This existing body of work highlights the potential of signal processing and machine learning methods to construct accurate classifiers of background EEG. Despite this significant progress, more can be achieved in this area. Thus far, progress has been confined to individual research groups pursuing different approaches. Comparing methods is difficult for many reasons^17^, including the lack of an accepted standard grading scheme^3^ and freely-available EEG data. Aiming to address some of these limitations—and inspired by the success of an open-access neonatal EEG data set with annotations^18^—we present an open-access EEG data set recorded within the first days after birth for infants with a HIE diagnosis. Multiple 1-hour EEG epochs for each infant were graded for severity of background abnormality. This data could be used to develop new algorithms or benchmark existing ones. The data could also be used to assist in training of the review of background neonatal EEG.

## Methods

### Patients

A subset of EEG records were retrieved from data collected during a medical-device trial. The clinical investigation evaluated the effectiveness of a machine-learning algorithm to detect seizures^19,20^. Neonates that were clinically determined to be at-risk of seizures, with a gestational age between 36 to 44 weeks, and admitted to the neonatal intensive care unit (NICU) were considered for inclusion in the study. After written and informed consent from a guardian or parent, neonates were enrolled over a period from January 2011 to Feburary 2017. Data were collected across 8 neonatal centres in Ireland, the Netherlands, Sweden, and the UK.

As part of the medical-device trial, 472 neonates were recruited^20^. From this group, 284 neonates were selected with a clinical HIE diagnosis and a valid EEG recording of at least 6 hours in duration. Eighteen infants were excluded because of a combined diagnosis, 68 were held out for a future validation set for development of an EEG algorithm, and 17 were excluded because the EEG did not start within 48 hours after birth. From the remaining 181, we only included 54 neonates for which we had permission to share the data. That is, the EEGs that were recorded in Cork University Maternity Hospital, Ireland. After closer examination of the EEG, a further neonate was excluded due to the low-quality of the EEG recording. For this cohort of 53 neonates, the median gestational age was 40 weeks, most were male (62%), and most (58%) received therapeutic hypothermia, as presented in Table 1.

**Table 1:** Clinical characteristics.

	*n* = 53
Gestational age (weeks)	40.0 (39.4 to 40.7)
Birth weight (g)	3,470 (3,190 to 3,800)
Sex (male)	33 (62%)
Sarnat score at 24 hours: ^†^	
mild	23 (43%)
moderate	18 (34%)
severe	8 (15%)
Therapeutic hypothermia:	
cooled	31 (58%)

Data represented as median (interquartile range) or number (%).

^†^*n* = 49

The study to collect EEG data at Cork University Maternity Hospital was approved by the Cork Ethics Research Committee. The same ethics committee also approved the Open Access release of the fully and irrevocably anonymised EEG recordings. Permission to share the data was obtained from the Data Protection Officer at University College Cork, Ireland.

### EEG

EEG was recorded as soon as possible after birth for a prolonged period up to 100 hours after birth. Two EEG machines were used, the NicoletOne ICU Monitor (Natus, Middleton, WI, USA) for 24 neonates and the Neurofax EEG-1200 (Nihon Kohden, Tokyo, Japan) for 29 neonates. EEG was sampled at a rate of 256 Hz (NicoletOne) and 200 Hz (Neurofax). Disposal electrodes were placed over the central (C3 and C4), frontal (F3 and F4), occipital (O1 and O2), and temporal (T3 and T4) regions and at the midline (Cz), using a reduced version of the 10:20 international system^20^. EEG was recorded relative to a reference channel: an average between C3 and C4 for the Neurofax recordings and FCz, a mid-line placement between Fz and Cz, for the NicoletOne recordings.

**Fig. 1:**
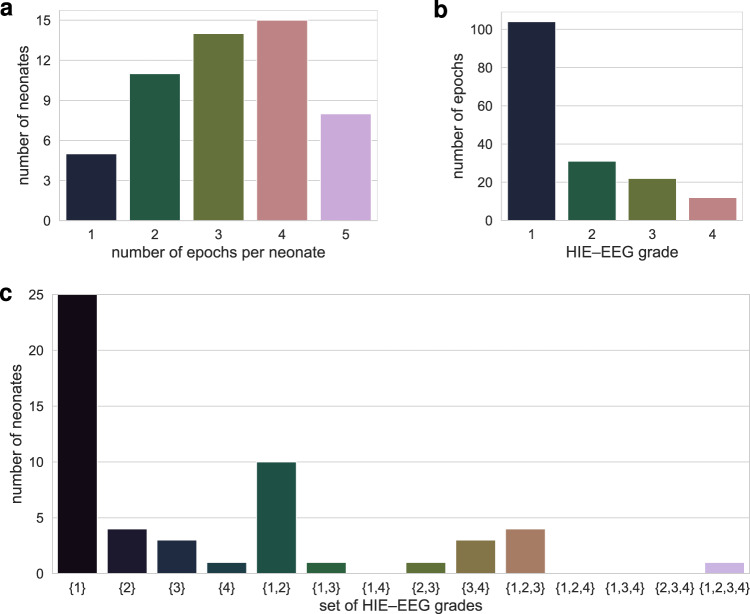
Distribution of EEG epochs. One-hundred and sixty nine  1-hour epochs were pruned from continuous EEG recordings from 53 neonates. Distribution of epochs per baby in (**a**) and grades of EEG hypoxic-ischaemic encephalopathy (HIE) in (**b**). Some neonates have more than one grade throughout the epochs: (**c**) illustrates the distribution of all possible combinations of sets of grades per neonate.

EEG was exported from the proprietary format of the NicoletOne and Neurofax machines to the open European Data Files (EDF) format and securely stored for off-line analysis. All data was fully anonymised. For each neonate, a maximum of 5 1-hour epochs were pruned from the continuous EEG recording. Artefacts are not uncommon in long-duration EEG recorded in a busy intensive care environment. Epochs were selected to avoid as much artefact as possible and for all epochs the majority of the epoch was artefact free. They were distributed in time throughout the duration of continuous recording but limited to the first 48 hours after birth. In total 169 epochs, exactly 60 minutes in duration, were included in the data set. The median number of epochs per neonate was 3, with an inter-quartile range of 2 to 4. Figure 1a illustrates the distribution of epochs per neonate.

**Fig. 2:**
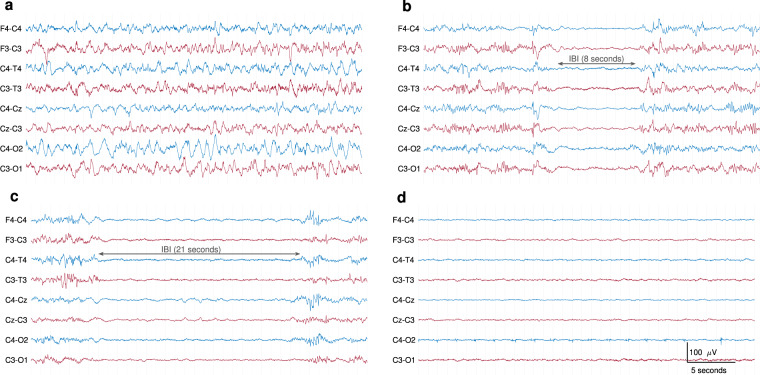
Examples of different EEG grades. Thirty-six seconds of EEG from different neonates. (**a**) normal or mildly abnormal EEG (grade 1); (**b**) moderately abnormal EEG (grade 2); (**c**) major abnormalities (grade 3); (**d**) inactive EEG (grade 4). Inter-burst intervals (IBI) are annotated in the grade 2 and grade 3 examples. All EEGs are in bipolar montage, plotted with the same time and amplitude scale, and bandpass filtered from 0.3 to 35 Hz.

Two clinical physiologists with expertise in neonatal EEG (authors SRM and GBB) independently graded each epoch using a commonly-used EEG classification scheme^6,8,9,16^. Table 2 provides an outline of this grading system. Where grades disagreed between the 2 experts, they jointly reviewed the epoch and decided on a consensus grade. The grading system includes measures of varying degrees of discontinuous activity, normal and abnormal patterns, symmetry and synchrony of activity across the hemispheres, and the quality or lack of sleep–wake cycling. Full details of the grading system can be found in Murray *et al*.^6^. Although seizures are not part of the grading system, some epochs did contain short-duration seizures. Due to the short-duration nature of these seizures comparative to the 1-hour epoch, there was sufficient background activity to assign a grade that was not solely based on the presence of seizures. Grade 0 (normal EEG) and grade 1 (mildly abnormal EEG) were combined into a new grade 1, to represent both normal and mildly abnormal EEG. The distribution of these 4 grades for the 169 epochs is illustrated in Fig. 1b. The vast majority of epochs are grade 1: 104 for grade 1, 31 for grade 2, 22 for grade 3, and 12 for grade 4. Not all neonates had the same grade throughout all epochs, as illustrated in Fig. 1c. The most common set of grades was {1} (*n* = 25), followed by {1, 2} (*n* = 10) and {1, 2, 3} (*n* = 4). One neonate had all 4 grades across 5 epochs. Example EEG segments for each grade are presented in Fig. 2.

**Table 2:** EEG Classification Adapted from Murray *et al*.^6^.

EEG grade (description)	Background	IBI (s)	Features of the EEG	Sleep–wake cycle
0 (normal)	Continuous	—	Normal physiologic features (e.g. anterior slow waves)	
1 (mild abnormalities)	Continuous	—	Slightly abnormal activity (e.g. mild asymmetry, mild voltage depression)	Or poorly defined
2 (moderate abnormalities)	Discontinuous	<10	Or clear asymmetry or asynchrony	Not clearly defined
3 (major abnormalities)	Discontinuous	10–60	Severe attenuation of background patterns	Or absent
4 (inactive)	Severe discontinuity	>60	Or background activity of <10 μV	Absent

IBI: inter-burst interval.

## Data Records

The EEG data with grades for each epoch are available at Zenodo (10.5281/zenodo.7477575)^21^. Data are provided as EDF files and as compressed comma separated values (CSV) files. The EDF files are stored in the EDF_format/ folder and the CSV files are stored in the CSV_format/ folder. Each 1-hour epoch is stored as a separate file, using the file name convention IDXX_epochY. For example, file ID10_epoch3 is the 3rd epoch for baby 10. A separate file called eeg_grades.csv, in CSV format, contains the grades assigned to each epoch. Another CSV file (metadata.csv) contains additional information on the epochs: a description of the quality of the EEG, whether seizures are present or not, reference electrode used in the recording, and the sampling frequency.

## Technical Validation

**Fig. 3:**
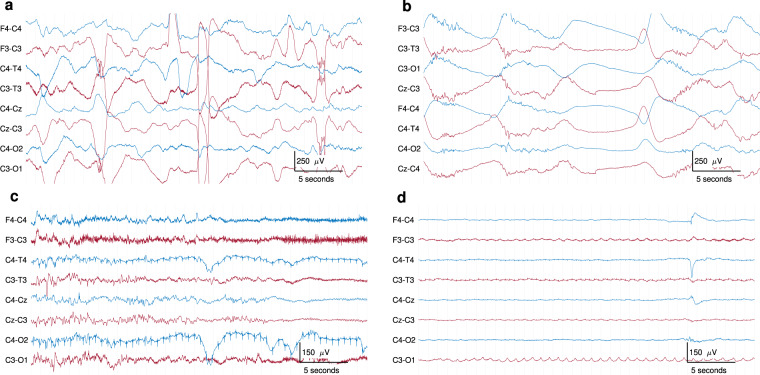
Examples of some typical EEG artefacts. Thirty-six seconds of EEG from different neonates. EEG segment with (**a**) high-amplitude movement artefact (grade 1 EEG); (**b**) sweat artefact (also grade 1 EEG);(**c**) ECG artefact on C4–O2 and C4–T4 and high-frequency muscle artefact, most prominent on F3–C3 (grade 3 EEG); (**d**) respiration artefact across the left hemisphere channels, clearly visible on C3–O1 (grade 4 EEG). All EEGs are in bipolar montage and bandpass filtered from 0.1 to 35 Hz.

EEG was recorded according to clinical standards in the NICU. The epochs of EEG were selected to be of high quality, with reduced artefact. However, as these EEGs are recorded in a busy intensive-care environment, they are not completely free from artefact. Figure 3 shows an example of some artefacts. The types of artefacts vary from biological origin, such as sweat artefacts, muscle, or respiration; to artefacts of non-biological origin, such as 50 Hz power-supply interference from nearby devices. Periodic checks of the electrode impedance causes a pause of EEG recording, resulting in periods of flat, near-zero EEG. The median duration of flat trace in the 60-minute epochs was 0.7 seconds, with an interquartile range of 0.4 to 1.0 seconds and a range of 0 to 431.1 seconds.

As part of a quality check of the EEG recording, we compare EEG power at lower and higher frequencies. The lower-frequency activity is a measure of cortical activity whereas the higher-frequency is unlikely to measure cortical activity and more likely to be nothing more than the noise floor^18^. We calculate power per channel across the bipolar montage F3–C3, T3–C3, O1–C3, C3–Cz, Cz–C4, F4–C4, T4–C4, and O2–C4. Each channel is bandpass filtered with an infinite-impulse response (IIR) filter, a type II Chebyshev filter of order 21. Power is then calculated within the passbands 0.5 to 16 Hz (low frequency) and 77 to 99 Hz (high frequency). The median power, over all epochs, for the low-frequency band is 175.4 μV^2^ (interquartile range, IQR: 83.7 to 419.8 μV^2^) and 8.4 μV^2^ (IQR: 1.6 to 36.2 μV^2^) for the higher frequency band. Thus we find that our estimates of the noise floor is considerably lower than the recording of EEG cerebral activity.

**Fig. 4:**
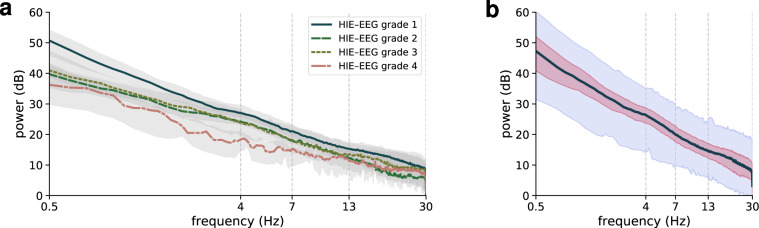
Spectra plotted on a log–log scale from epochs for each grade (**a**) and a grand-average for all epochs (**b**). Thick lines represent the median value across all epochs, and shaded areas represent the inter-quartile range in (**a**) and (**b**) and 95-th centile range in (**b**). There are 104 epochs for grade 1, 31 for grade 2, 22 for grade 3, and 12 for grade 4.

Next, to further validate the technical quality of the EEG, we computed the frequency response for all epochs and generated a set of quantitative EEG (qEEG) features^22,23^. Power spectral densities (PSD) were estimated using the Welch method with an 8-second Hamming window and 75% overlap. PSDs were calculated per channel using the same bipolar montage described previously. Each channel’s estimate is then averaged over all 8 channels for the 1-hour epoch. Figure 4 summarises the PSDs for all epochs per grade. Grades 1 to 3 indicate a linear log–log frequency response, known as a power-law response, in keeping with current understanding of neonatal EEG^10,22,24,25^. For grade 4, the response appears more nonlinear, but the lower number of epochs in this group (12, compared with 23, 31, and 105) may be a factor here.

The qEEG feature set consisted of 5 features: spectral power, range-EEG (rEEG), interhemispheric coherence, fractal dimension, and spectral edge frequency^22,23^. Features are estimated using the same bipolar montage described previously. Spectral power and coherence features are generated separately in 4 frequency bands: delta (0.5–4 Hz), theta (4–7 Hz), alpha (7–13 Hz), and beta (13–30 Hz). The rEEG is calculated within the 1–20 Hz bandwidth and assessed at the lower-, median-, and upper-margins^23^. Inter-hemispheric coherence is an averaged value of coherence calculated between the following channel pairs: F3–C3 and F4–C4, T3–C3 and T4–C4, and O1–C3 and O2–C4. Spectral power, coherence, fractal dimension, and spectral edge frequency (95%) are estimated on a 64 second segment of EEG with 50% overlap. The median value of all segments is used to summarise the feature over the 1-hour epoch. All features, excluding coherence, are estimated on a channel by channel basis and summarised by the median value across channels. Features were generated using the NEURAL toolbox (https://github.com/otoolej/qEEG_feature_set, version 0.4.4).

**Fig. 5:**
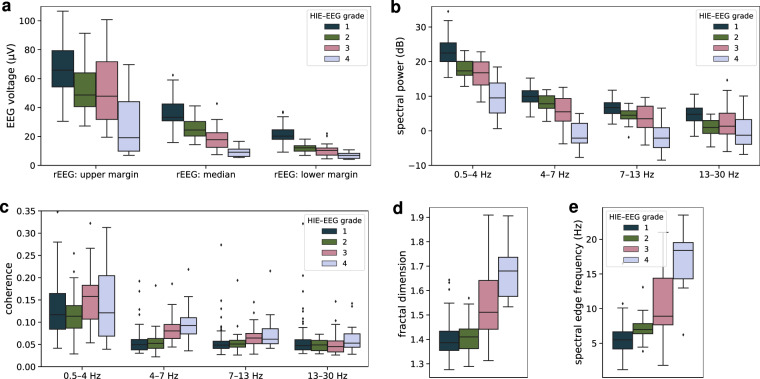
Quantitative summary measures of the EEG for the 4 HIE–EEG grades. Three summary measures of the range-EEG (rEEG) in (**a**), spectral power in (**b**), inter-hemispheric coherence in (**c**), fractal dimension in (**d**), and spectral edge frequency in (**e**). Spectral power and coherence features are calculated for 4 different frequency bands.

Figure 5 plots the distribution of the 5 features, highlighting the differences for many features across the 4 grades. The rEEG in Fig. 5a, a measure of peak-to-peak voltage, shows decreasing EEG amplitude through the 4 grades, with the difference between the grades particularly pronounced in the median rEEG feature. Similarly, the difference in spectral power for the 4 grades across the 4 frequency bands is evident in Fig. 5b. Significant, although low-valued, interhemispheric coherence is present across the 4 frequency bands, with higher levels of coherence in the delta (0.5–4 Hz) band compared to the other 3 frequency bands. Both the fractal dimension and spectral edge frequency plots indicate a difference in spectral shape for the grades, in the form of a decreasing slope of the log–log spectra with increasing grades.

## Usage Notes

The EEG files are stored in both EDF and CSV format. The EDF format was developed in 1992 for sleep EEG files and has remained a standard open-format for EEG files^26^. EDF files can be viewed in most EEG review software, including free versions such as *EDFbrowser* (https://www.teuniz.net/edfbrowser/). The format stores data in 16-bit integers and therefore will likely be converted to 64-bit floating-point numbers before analysis or viewing of the data. Storing EEG in 16-bit integers reduces the file size comparative to 64-bit floating-point numbers but does so in a lossy manner; however many lossless compression algorithms can now match this compression without loss of information. Despite its outdated structure, it still remains a standard open format for EEG review.

For analysis using software tools, special libraries are required to read EDF files as this format is not used for other data types. For this reason, to simplify the process of loading the data for analysis we also provide the widely accessible CSV format. Our CSV format stores time (in seconds) and voltage (in micro-Volts) at each of the 9 channels. The first line of each CSV file contains a header with the name of each column. These files are compressed using the cross-platform XZ format, which uses the Lempel–Ziv–Markov chain algorithm. Freely available compression tools can be used to decompress the files. For example, 7z (https://www.7-zip.org/) for Windows operating systems or XZ Utils (https://tukaani.org/xz/) for Linux operating systems. Alternatively, many programming languages can provide this decompression when importing the data. In the Python programming environment (Python Software Foundation, https://www.python.org/) for example, the read_csv function from the Pandas package can directly read in the tabular data compressed with XZ^27^:


import pandas as pdeeg_df = pd.read_csv(“ID10_epoch3.csv.xz”)


for example file ID10_epoch3.csv.xz. Likewise, the R programming environment (R Core Team, https://www.R-project.org/), the read.csv function can directly import data in the compressed XZ format.


eeg_df < - read.csv(“ID10_epoch3.csv.xz”)


For Matlab (The Mathworks, Inc., United States) and Julia (The Julia Project, https://julialang.org/)^28^, the CSV files must be uncompressed before importing. In Matlab,

eeg_tb = readtable(“ID10_epoch3.csv”);where ID10_epoch3.csv is the uncompressed version of ID10_epoch3.csv.xz. In Julia,


using CSVusing DataFrameseeg_df = CSV.read(“ID10_epoch3.csv”, DataFrame)


The data could be used for training purposes. For this, the data can be viewed in an EEG viewer using both a referential or bipolar montage. Data are provided in the raw referential format for processing purposes, however visual analysis is typically conducted using a bipolar montage. The bipolar montage displayed during recording and used for background scoring contains the following bipolar electrode pairs: F4–C4, C4–O2, F3–C3, C3–O1, T4–C4, C4–Cz, Cz–C3, C3–T3. This contains both antero–posterior and transverse elements. Typical display settings for reviewing neonatal EEG include: sensitivity 70–100 μV/cm, timebase 15–20 mm/sec, and bandpass filter 0.5–70 Hz. Amplitude-integrated EEG (aEEG) channels are popular in clinical review and within this montage might include aEEG channels for F4–C4, F3–C3 and C4–C3.

The EEG data could also be used to develop an automated EEG grading algorithm. These classification algorithms use signal processing and machine-learning methods to extract information from the EEG that is characteristic of the particular grade of EEG^8–17^. The first stage in algorithm development is to preprocess the data. This will include a bandpass filter, typically followed by downsampling. The bandpass filter, at 0.5 to 30 Hz for example, removes the 50 Hz power-line noise and very-slow activity (<0.5 Hz) often associated with artefact such as DC drift or sweat artefact. The bandpass filter also allows for downsampling without aliasing. Downsampling is commonly applied to reduce the algorithm’s computational load with negligible loss in performance. The preprocessed EEG is then ready for use in a classifier to grade the EEG.

There are 2 approaches to developing a grading algorithm which incorporate machine-learning models. The first is to use signal-processing methods to extract a set of features from the preprocessed EEG and then combine these features using a machine-learning model^8–15,17^. For this approach, we must develop and curate a set of features that adequately generalises the main discriminating factors among the 4 grades. As Fig. 5 indicates, there are potentially many different features that could discriminate, with varying levels of accuracy, between the 4 grades. The second approach is to use deep-learning methods, which provides an end-to-end (EEG to grade) solution^16,29^. These methods automatically extracts and combines the features in a single neural network. For this approach, we must the select the type of neural network to use, for example a convolutional or recurrent neural network, and then design the specific architecture of the network.

For both approaches, the machine-learning model is constructed using a data-driven approach through training and testing. We recommend training and testing the model using some form of cross validation, ideally leave-one-out. The split of training and testing data should be done on a neonate level, not on an epoch level. This will avoid testing a model that was trained using epochs from the same neonate. Regardless of which approach is used, there is, unfortunately, no one-size-fits-all model. That is, different applications will require different models. Considerable care and attention to the design process is required to develop an accurate and robust classifier, but certainly worth the effort given the potential clinical utility of such an algorithm to improve health outcomes for infants with HIE.

## Data Availability

Custom code was not used to generate the data. EEG files were exported from proprietary format to EDF files using the associated EEG reviewing software for the NicoletOne and Neurofax EEG machines. Details on how to view the EEG data and import it into programming environments is described in the Usage Notes section. To assist with computer-based analysis of the EEG, we provide freely-available code to downsample the EEG to a lower and uniform sampling rate. For quantitative or machine-learning analysis, the neonatal EEG is often downsampled to a lower sampling rate, as the majority of the power is typically below 10 to 20 Hz. For example, Fig. 5e shows that 95% of spectral power is below 25 Hz. The processing routines include an anti-aliasing filter before downsampling. Both Matlab and Python versions of the code are included at https://github.com/otoolej/downsample_open_eeg (commit: 22e92db).

## References

[1] Kurinczuk JJ, White-Koning M, Badawi N (2010). Epidemiology of neonatal encephalopathy and hypoxic-ischaemic encephalopathy. Early Hum. Dev..

[2] Perez A (2013). Long-term neurodevelopmental outcome with hypoxic-ischemic encephalopathy. J. Pediatr..

[3] Walsh BH, Murray DM, Boylan GB (2011). The use of conventional EEG for the assessment of hypoxic ischaemic encephalopathy in the newborn: a review. Clin. Neurophysiol..

[4] Holmes G (1982). Prognostic value of the electroencephalogram in neonatal asphyxia. Electroencephalogr. Clin. Neurophysiol..

[5] Pressler RM, Boylan GB, Morton M, Binnie CD, Rennie JM (2001). Early serial EEG in hypoxic ischaemic encephalopathy. Clin. Neurophysiol..

[6] Murray DM, Boylan GB, Ryan CA, Connolly S (2009). Early EEG findings in hypoxic-ischemic encephalopathy predict outcomes at 2 years. Pediatrics.

[7] Tagin MA, Woolcott CG, Vincer MJ, Whyte RK, Stinson DA (2012). Hypothermia for neonatal hypoxic ischemic encephalopathy: an updated systematic review and meta-analysis. Archives of Pediatrics and Adolescent Medicine.

[8] Korotchikova I, Stevenson NJ, Walsh BH, Murray DM, Boylan GB (2011). Quantitative EEG analysis in neonatal hypoxic ischaemic encephalopathy. Clin. Neurophysiol..

[9] Ahmed R, Temko A, Marnane W, Lightbody G, Boylan G (2016). Grading hypoxic-ischemic encephalopathy severity in neonatal EEG using GMM supervectors and the support vector machine. Clin. Neurophysiol..

[10] Stevenson NJ (2013). An automated system for grading EEG abnormality in term neonates with hypoxic-ischaemic encephalopathy. Annals Biomed. Eng..

[11] Raurale, S. A. *et al*. Tracé alternant detector for grading hypoxic-ischemic encephalopathy in neonatal EEG. In *29th Eur. Signal Process. Conf*., 1177–1181, (IEEE 2021).

[12] Raurale, S. A., Nalband, S., Boylan, G. B., Lightbody, G. & O’Toole, J. M. Suitability of an inter-burst detection method for grading hypoxic-ischemic encephalopathy in newborn EEG. In *41st Int. Conf. IEEE Eng. Med. Biol. Soc*., 4125–4128, (IEEE 2019). 10.1109/EMBC.2019.8857000 31946778

[13] Matic V (2014). Holistic approach for automated background EEG assessment in asphyxiated full-term infants. J. Neural Eng..

[14] Matic V (2015). Improving reliability of monitoring background EEG dynamics in asphyxiated infants. IEEE Trans. Biomed. Eng..

[15] Guo J, Cheng X, Wu D (2020). Grading method for hypoxic-ischemic encephalopathy based on neonatal EEG. Computer Modeling in Engineering and Sciences.

[16] Raurale SA (2021). Grading hypoxic-ischemic encephalopathy in neonatal EEG with convolutional neural networks and quadratic time–frequency distributions. J. Neural Eng..

[17] Moghadam SM (2021). Building an open source classifier for the neonatal EEG background: a systematic feature-based approach from expert scoring to clinical visualization. Front. Human Neurosci..

[18] Stevenson NJ, Tapani K, Lauronen L, Vanhatalo S (2019). A dataset of neonatal EEG recordings with seizure annotations. Sci. Data.

[19] Pavel AM (2020). A machine-learning algorithm for neonatal seizure recognition: a multicentre, randomised, controlled trial. Lancet Child Adolesc. Heal..

[20] Pavel AM (2021). Neonatal seizure management: is the timing of treatment critical?. J. Pediatr..

[21] O’Toole JM (2022). Zenodo.

[22] Finn D, O’Toole JM, Dempsey EM, Boylan GB (2019). EEG for the assessment of neurological function in newborn infants immediately after birth. Arch. Dis. Child. Fetal Neonatal Ed..

[23] O’Toole JM, Boylan GB (2019). Quantitative preterm EEG analysis: the need for caution in using modern data science techniques. Front. Pediatr..

[24] Korotchikova I (2009). EEG in the healthy term newborn within 12 hours of birth. Clin. Neurophysiol..

[25] Stevenson NJ, Mesbah M, Boylan GB, Colditz PB, Boashash B (2010). A nonlinear model of newborn EEG with nonstationary inputs. Annals Biomed. Eng..

[26] Kemp B, Värri A, Rosa AC, Nielsen KD, Gade J (1992). A simple format for exchange of digitized polygraphic recordings. Electroencephalogr. Clin. Neurophysiol..

[27] The pandas development team (2022). Zenodo.

[28] Bezanson J, Edelman A, Karpinski S, Shah VB (2017). Julia: A fresh approach to numerical computing. SIAM Review.

[29] Raurale, S. A., Boylan, G. B., Lightbody, G. & O’Toole, J. M. Grading the severity of hypoxic-ischemic encephalopathy in newborn EEG using a convolutional neural network. In *42nd Int. Conf. IEEE Eng. Med. Biol. Soc*., 6103–6106, (IEEE, 2020). 10.1109/EMBC44109.2020.9175337 PMC7613058 33019363

